# Review of protein structure-based analyses that illuminate plant stress mechanisms

**DOI:** 10.1016/j.csbj.2025.07.021

**Published:** 2025-07-13

**Authors:** Fatima Shahid, Neeladri Sen, Hawa Najibah Rasni, Nurulhikma Md Isa, Nyuk Ling Ma, Christine Orengo, Su Datt Lam

**Affiliations:** a Department of Applied Physics, Faculty of Science and Technology, Universiti Kebangsaan Malaysia, Bangi 43000, Malaysia; b Institute of Structural and Molecular Biology, Division of Biosciences, University College London, London, England WC1E 6BT, United Kingdom; c Department of Biological Sciences and Biotechnology, Faculty of Science and Technology, Universiti Kebangsaan Malaysia, Bangi 43000, Malaysia; d BIOSES Research Interest Group, Faculty of Science & Marine Environment, Universiti Malaysia Terengganu, 21030, Malaysia; e Center for Global Health Research (CGHR), Saveetha Medical College, Saveetha Institute of Medical and Technical Sciences (SIMATS), Saveetha University, Chennai, India

**Keywords:** AlphaFold2, AlphaFold3, Plant stress, Protein structures, Structural analysis

## Abstract

Plants face formidable challenges due to environmental stresses, including pathogens, salt, drought, cold, heat, heavy metal exposure, and flooding, all of which affect growth and agricultural productivity. To combat these stresses, plants have evolved various adaptive mechanisms, including the expression of stress-response proteins. Exploring the three-dimensional structures of plant proteins can be valuable for discovering and characterising stress tolerance mechanisms at the molecular level. Until recently, large-scale analyses were not feasible due to the limited number of experimentally determined plant protein structures. However, the recently developed AlphaFold, RoseTTA-Fold, and ESM-fold protein structure prediction methods, along with their associated portals, now provide hundreds of millions of high-quality predicted 3D models, covering a wide range of plant proteins. This review highlights insights from recent structural investigations into plant stress response using experimental or predicted protein structures. We include analyses of diverse paralogs and isoforms and insights from molecular docking and molecular dynamics simulations. We consider the value of using experimental and predicted structural data in understanding the mechanisms of common stress-modulating plant proteins. Studying the structures of these proteins together with their inferred functions can aid improvements in crop productivity, help foster sustainable agriculture, and contribute to global food security efforts.

## Graphical Abstract

**Figure:**
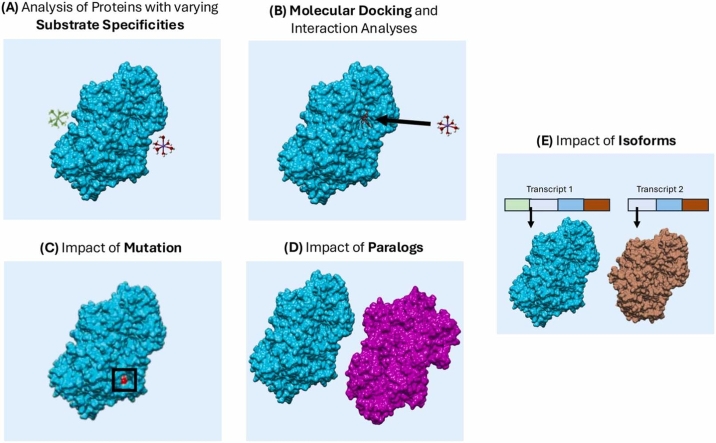

## 1 Introduction

Environmental stress factors, including salt, drought, cold, heat, heavy metal exposure, and flooding, pose significant challenges to plant growth and agricultural productivity [1], [2]. For instance, soil salinity presents a severe threat, and is likely to render ∼50 % of cultivable land unusable by 2050, with over 1125 million hectares of global arable land already affected [3]. Plants counter these stresses via various mechanisms, including osmotic pressure regulation, osmolyte production, and activation of specific genetic pathways targeting ion transporters [4]. Similarly, biotic stress involves pathogens (bacteria, fungi, viruses), pests, and parasites, exploiting them for nutrients and affecting plants by secreting toxins [5]. Nonetheless, plants have adapted to tackle such stress factors by employing a plant immune system with physical barriers like wax, cuticles, and trichomes as the first line of defence, and by producing chemicals that kill attacking pathogens. Also, by expressing receptors that recognize and stimulate immune pathways to destroy pathogens effectively [6].

Plant proteins are essential biomolecules that govern vital physiological processes for growth, adaptation, and defence. They play diverse roles, from facilitating photosynthesis and nutrient transport to orchestrating defence responses and signal transduction. A comprehensive understanding of plant proteins is important for enhancing crop productivity, promoting sustainable agriculture, and addressing global food security challenges. To gain deeper insights into the functions of stress-related plant proteins, researchers have exploited protein structure modelling in order to identify key residues involved in plant adaptation, thereby gaining insights into the role of these proteins in stress signal transduction and regulatory networks [7].

This review explores the value of protein structures for illuminating the 3D architecture and interactions of plant proteins involved in stress mediation. We examine how structural studies have contributed over the recent years and how structural investigations can increase the knowledge of plant mechanisms to drive crop improvement and enable targeted interventions for enhanced plant resilience and productivity.

## 2 Current extent of experimentally solved plant protein structures and examples of structural analyses performed to investigate the mechanisms of plant stress response

The advent of high-throughput sequencing techniques has vastly expanded protein sequence repositories and revolutionized our understanding of sequence diversity, opening new avenues for comprehending protein functions. However, a significantly smaller number of protein structures have been experimentally characterised. The scientific community has endeavoured to address this gap by continuously improving structure determination. For example, significant developments in cryo-electron microscopy (cryo-EM) to solve the structures of small complexes and yield high-resolution models have been important landmarks. Modern electron detectors can now capture images at resolutions below 4 Å [8]. Moreover, crystallography remains a primary technique to precisely determine the three-dimensional structure of proteins [9]. Nevertheless, the number of experimentally solved protein structures in the Protein Data Bank (PDB) is only 229,183 (out of a total of 254,254,987 sequences in UniProt, as of 24th December 2024) [10], [11]. This number further declines when considering plant structures. For instance, as of 24th December 2024, there are only 5393 experimentally derived plant structures, with 1372, 106, 111, and 145 belonging to *Arabidopsis thaliana* (Arabidopsis), *Glycine max* (soybean), *Oryza sativa* subsp. *Japonica* (rice) and *Zea mays* (maize), respectively. However, the number of protein sequences deposited in the UniProt database (as of 24th December 2024) is 136,331 for Arabidopsis, 85,132 for soybean, 148,883 for rice, and 85,772 for maize.

Despite this disparity between the abundance of protein sequences and the limited availability of experimentally determined structures, recent near-accurate deep learning based structure prediction tools such as AlphaFold2, AlphaFold3, RoseTTA-Fold, and ESM-fold have markedly altered this landscape [12], [13], [14], [15]. These transformative approaches have provided hundreds of millions of good-quality quality 3D-models offering a more comprehensive understanding of protein structures and the functional insights they bring. The benefits slowly accruing from this expansion in structural data are discussed in later sections of this review.

In this section, we highlight specific examples of analyses using experimental structures that have advanced our understanding of the mechanisms underlying stress responses.

### 2.1 Structural analyses of substrate and ligand specificities in plant stress response proteins

Proteins interact with their substrates or ligands to carry out their functions; hence, the precise specificity of a protein for its substrate or ligand is vital. Structural analysis of protein features, particularly of the shape and arrangement of residues and their physico-chemical properties, in the binding site, can help in understanding substrate or ligand specificity.

**Fig. 1:**
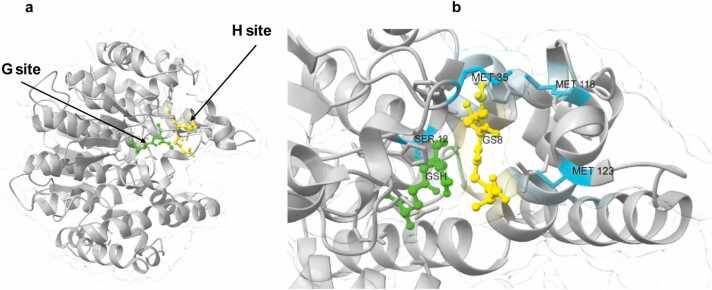
The crystal structure of reduced GST is shown in grey (PDB: 6ezy). a) The glutathione binding site (G-site) and hydrophobic substrate-binding site (H-site) are indicated with arrowheads pointing to the bound ligands. Glutathione (GSH) is bound at the G-site, and sulfenylated glutathione (GSOH) is present in the H-site. b) Key residues, including catalytic serine (S12) and methionines (M35, M118, M123), are highlighted in Cyan. The PDB structure has been visualized using UCSF Chimera [20]. (For interpretation of the references to color in this figure legend, the reader is referred to the web version of this article.)

For example, plant Glutathione Transferases (GSTs) exhibit promiscuity, enabling them to act on multiple substrates. GSTs play a role in mediating xenobiotic detoxification of herbicides containing toxic compounds by conjugating them to glutathione (GSH). Structurally, all GSTs consist of two distinct domains: the N-terminal domain adopts a thioredoxin-like fold containing both α-helices and β-sheets, and houses the conserved G-site, which is hydrophilic and binds GSH. The C-terminal domain, composed primarily of five or more α-helices, forms the H-site, which is hydrophobic and interacts with diverse toxic electrophilic molecules (Fig. 1a) [16].

The G-site features key residues such as serine, tyrosine, or cysteine that stabilize the thiolate anionic form of GSH by lowering its pKa, thereby enabling it to act as a strong nucleophile. In contrast, the H-site binds hydrophobic electrophilic substrates such as 2,4-dinitrochlorobenzene (CDNB) or lipid peroxides [17], [18]. Structural variation in the H-site among different GST isoforms supports broad substrate specificity. The H-site typically contains hydrophobic residues that position the substrate near the activated GSH for optimal catalysis. The functionality of GSTs is intricately linked to their three-dimensional structure, especially the spatial configuration and dynamics of the G- and H-sites (Fig. 1a). Proper alignment of these domains is critical for substrate recognition, nucleophilic attack, and stabilisation of the transition state.

In some GSTs, the structural loops near the active site, such as the β2–α2 loop (in which Met35 is located) and the α4–α5 loop (in which Met118 and Met123 are located), are crucial for diverse substrate binding (Fig. 1b). In the case of *Arabidopsis thaliana* GSTF9, oxidation of methionine residues located within these loops occurring under oxidative stress induces local flexibility, particularly affecting the H-site [19]. This structural change, which involves residues 120–127 in the α4–α5 loop, compromises the oxidized enzyme’s ability to bind and process hydrophobic peroxides such as tert-butyl hydroperoxide and cumene hydroperoxide. Although the peroxidase activity is altered, the stress response is maintained through the activation of a complementary reductase, restoring redox balance [18].

Another example of diversity in protein-ligand binding specificity is the stress-inducible lectins. These possess diverse structural folds that directly influence their ligand-binding specificity and biological function. Amongst the stress-inducible lectins, two major groups within the jacalin-related lectin (JRL) family can be distinguished based on their preference for galactose or mannose. These preferences are closely linked to differences in their structures and the organization of their carbohydrate-binding sites [21].

**Fig. 2:**
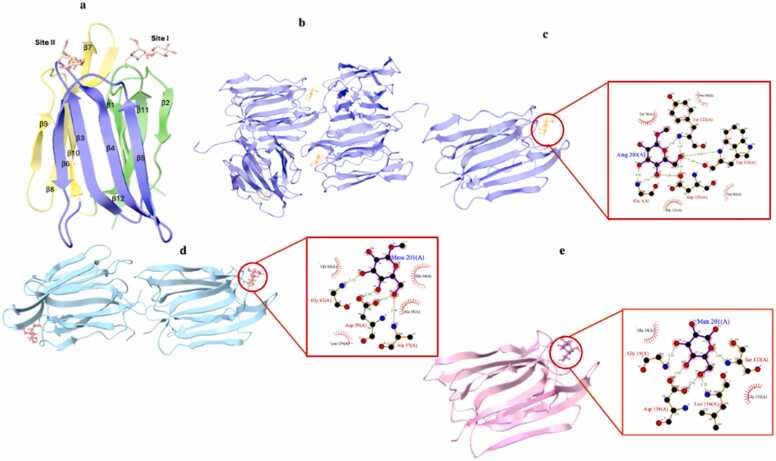
a) Ribbon representation of a JRL monomer, highlighting two separate carbohydrate-binding sites. The β-prism structure is displayed with its three Greek Key motifs individually coloured: green for subunit 1, blue for subunit 2, and yellow for subunit 3. (The three Greek key β-sheets are composed of numbered β-strands: (β1–2 + β11–12, β3–6, and β7–10) b) Tetrameric galactose binding lectin (PDB ID: 1JAC) c) Monomer from PDB ID 1JAC bound to galactose d) lectin from *Ananas comosus* (shown as a monomer) bound to mannose (PDB ID:6FLY) e) the same lectin from *Ananas comosus* (shown as a biologically active dimer) bound to methyl alpha-D-mannopyranoside (PDB ID: 6FLZ). The PDB structures were visualized using UCSF Chimera [20]. The zoom-ins show LIGPLOT [28] representations of the sugar binding interactions. (For interpretation of the references to color in this figure legend, the reader is referred to the web version of this article.)

The first type comprises the galactose-specific JRLs (gJRLs), typically forming tetramers of identical protomers. Each protomer adopts a β-prism fold, characterized by three Greek key motifs, each formed by antiparallel β-strands, arranged to create a three-fold symmetric prism, that is also shared by mannose-binding lectins (Fig. 2a). The sugar-binding site comprises Gly1, Phe47, Tyr78, Asp125, Tyr122, and Trp123 (Figs. 2b and 2c) [22]. A key interaction occurs between the amino group of Gly1, forming a hydrogen bond with the O4 atom of galactose. Aromatic residues, notably Tyr78, contribute to binding by stacking against the non-polar face of the galactose ring [23]. gJRLs preferentially bind the disaccharide Galβ(1→3)GalNAc, commonly found on the surfaces of fungi, bacteria, and viruses, suggesting a role in pathogen defence [24], [25].

The second JRL comprises the mannose-specific JRLs (mJRLs), such as those from jackfruit seeds (*Artocarpus integrifolia*). mJRLs can form dimers, tetramers, or octamers in solution and are commonly upregulated in response to environmental stresses, suggesting a crucial role in plant adaptation to conditions such as salinity, pathogen attack, herbivory, and hormone treatments (e.g., jasmonate and ABA) [26]. When oligomerising as a tetramer, they consist of four identical protomers comprising the β-prism fold, which is also observed in gJRLs [26]. However, mJRLs typically lack the post-translational proteolytic cleavage, which is observed in the gJRLs. This results in protomers made up of a single, unprocessed polypeptide chain, which contains an additional loop near the sugar-binding site, which restricts galactose access while enhancing specificity for mannose and oligomannosides [27]. Mannose binding in mJRLs is mediated by highly conserved amino acids located on two key loops adjacent to the β1–β2 and β11–β12 strands (Fig. 2a in green).

While most mJRLs contain a single sugar-binding site per protomer, certain lectins (such as those from pineapple (*Ananas comosus*) stem and banana) contain two distinct binding sites per protomer (Fig. 2a). Both sites can bind D-mannose and methyl-α-D-mannopyranoside. Structural studies revealed in site 1, both glycans bind to loops from the β1–β2 and β11–β12 strands of the first Greek key motif. D-mannose forms hydrogen bonds with multiple residues including Asp136, Ser133, Gly15, and Leu134 (Fig. 2d). For methyl-α-D-mannopyranoside most of these interactions occur, but the methyl group introduces steric hindrance, causing a 120° rotation in the hydrogen bond between its O5 atom and Ser133’s hydroxyl group (Fig. 2e) [24], [25], [26] to optimise the binding. Similar mechanisms are observed at Site 2.

#### 2.1.1 Structural analyses of plant proteins involved in immune responses

**Fig. 3:**
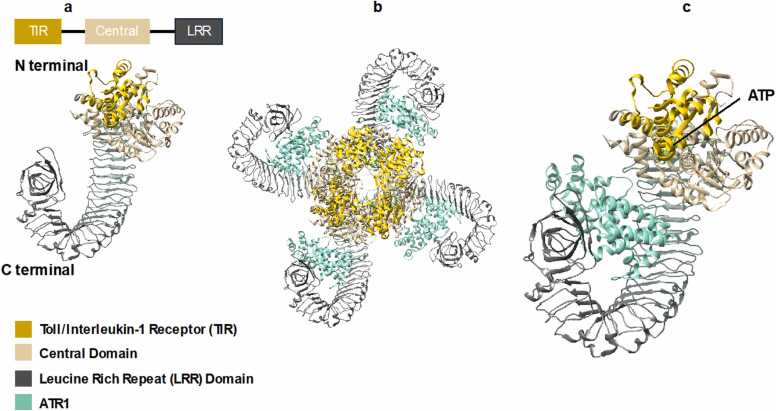
Structures of TIR NLR (RPP1, PDB ID: 7CRC) a) Domain composition of the RPP. b) Activated tetramer configuration RPP1 bound to Avirulence protein ATR1 c) Enlarged image of a monomeric unit from the active assembly shown in b (ligand binding site is labelled). [32].

**Fig. 4:**
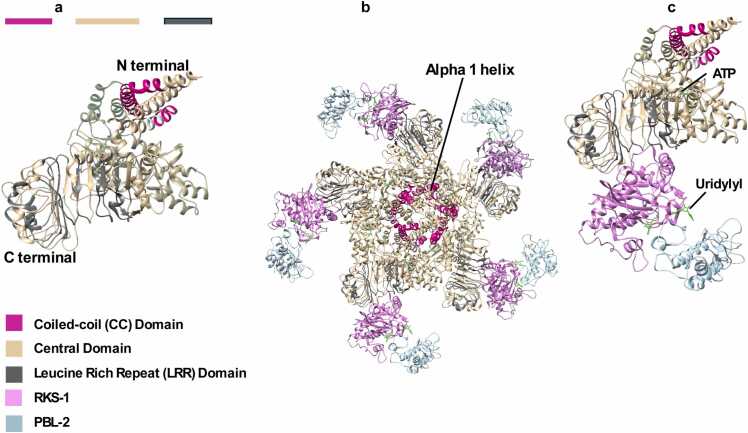
Structures of CC-NLR (ZAR1, PDB ID: 6J5T). a) Domain components of the Zar1 protein. b) Assembled pentameric complex bound to RKS1, PBL2 c) Enlarged image of a monomeric unit from the active assembly shown in b (ATP and Uridylyl binding sites are labelled) [32].

Plants resist pathogen attacks using innate immune response proteins, such as NLRs (Nucleotide-binding/leucine-rich repeat receptors). NLRs consist of three domains: an N-terminal domain, a central domain, and a C-terminal domain (Figs. 3a and 4a). Plant NLRs typically detect pathogen effectors through their C-terminal leucine-rich repeat (LRR) domains, mediating direct or indirect recognition. These LRR domains facilitate protein–protein interactions and are crucial for binding pathogen-derived molecules or sensing effector-induced modifications in host proteins. Based on their N-terminal domains, plant NLRs are classified into two major groups: Coiled Coil NLRs (CC-NLRs), which contain a coiled-coil domain, and Toll/interleukin-1 receptor NLRs (TIR-NLRs), which possess a Toll/interleukin-1 receptor (TIR) domain [29]. Upon recognition of pathogen effectors, conformational changes are triggered within the NLR protein, particularly the central nucleotide-binding domain (NBD), which acts as an ATPase that exchanges adenosine diphosphate (ADP) for adenosine triphosphate (ATP). This nucleotide exchange promotes oligomerization of NLRs, forming resistosome complexes that initiate downstream immune signalling pathways. These signalling cascades ultimately activate defence responses, including programmed cell death, to restrict pathogen proliferation and enhance plant innate immunity. NLRs can be comprised of various C-terminal domains that influence substrate specificity. The jelly roll/Ig-like domain (C-JID) is located immediately after the LRR portion, whereas other domains, such as ankyrin (ANK) repeats, tetratricopeptide (TPR) repeats, and integrated domains, represent alternative ligand-binding domains [30].

An inactive state of the NLR protein is maintained by inhibitory contacts, which include interactions between the C-terminal leucine-rich repeat (LRR) domain and components of the central domain (NBD). This closed, inactive conformation is typically associated with ADP bound to the nucleotide-binding pocket within the central domain [29].

. TIR-NLRs are activated by effector proteins binding directly to the LRR of the C-terminal domain (Figs. 3b and 3c). The N-terminal TIR domain in these NLRs is associated with Nicotinamide-Adenine Dinucleotidase (NADase) activity. In *Arabidopsis thaliana*, (Recognition of Peronospora Parasitica 1 RPP1 TIR-NLR) plays a crucial role in defending against *Peronospora parasitica*. When the plant encounters the pathogen, the pathogen releases effector proteins such as ATR1. RPP1 TIR-NLR binds ATR1 directly and forms a tetrameric resistome complex that triggers plant immune signalling pathways. Structural studies revealed that the pathogen effector induces a conformational change in the LRR of the RPP1 TIR-NLR C-terminal domain such that it bends around the effector and forms a clover-like shape. Wan *et al.* reported that the tetramer is structurally conserved and is required for NADase activity. This NADase reaction produces nicotinamide and variant-cyclic Adenosine Diphosphate Ribose ADPR (v-cADPR), a signalling molecule formed by glycosidic bonding of cleavage products of NAD, which generate signals to initiate EDS1-helper NLR-mediated immune pathways to trigger cell death [31]. The complex, therefore, acts as a signalling platform, coordinating the plant's immune defence against the invading pathogen [31].

Such structural insights highlight the diverse pathogen recognition strategies in plant NLRs to aid protein engineering for increased resistance [17]. The essentiality of NLRs in disease resistance has led to the creation of repositories like NLRscape, which contains more than 80,000 NLR protein sequences, including those essential for plant innate immunity. NLRscape enables users to carry out sequence and structure-based clustering, secondary structure analysis, and motif recognition [32].

Förderer *et al.* structurally characterized the disease resistance mechanism of ZAR1, a CC-NLR, that indirectly recognizes pathogens [33]. A helper protein binds the NLR (LRR domain), but the complex remains in an inactive form until a decoy protein modified by the pathogen effector protein is recognized by the helper protein. In ZAR1, the helper protein is a resistance kinase named RKS1. The pathogen *Xanthomonas campestris* releases an effector protein, AvrAC, which uridylylates a decoy plant protein called PBL2_UMP._ Uridylylated PBL2_UMP_ binds to RKS-1 bound to ZAR1, forming ZAR1-RKS1-PBL2_UMP_ (Figs. 4b and 4c). Biochemical studies have shown that this triggers an exchange of ADP with ATP in the central domain of ZAR1, leading to oligomerization of the ZAR1-RKS1-PBL2_UMP_ as an active pentamer which inserts into the cell membrane. Structural studies showed that in the oligomer the α1 helices of each of the five N-terminal domains of ZAR1 in the oligomer form a funnel-like structure through the membrane, facilitating the influx of secondary messenger calcium ions from outside the cell leading to cell death [29].

**Fig. 5:**
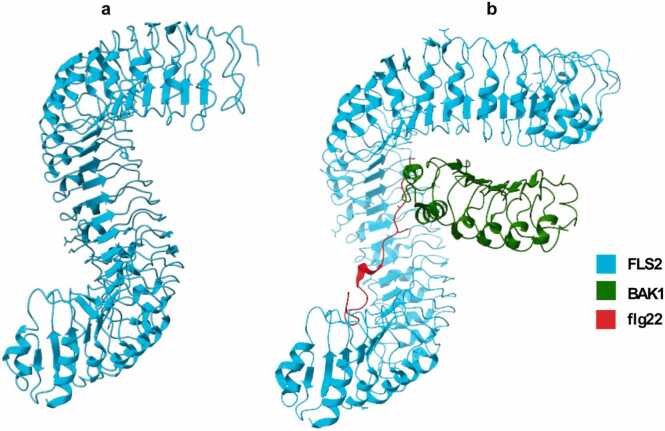
a) Twisted super helical structure FLS2 (PDB ID:4MNA) b) structural arrangement of FLS2, flg22, and BAK1 (PDB ID:4MN8).

Another type of immune modulating proteins in plants are the receptor-like kinases (RLKs), especially leucine-rich repeat receptor kinases (LRR-RKs). These proteins play crucial roles in plant stress signalling, with > 200 members identified in *Arabidopsis thaliana* [34]. Structurally, LRR-RKs consist of three key domains: an extracellular domain rich in leucine-rich repeats responsible for ligand recognition, a single-pass transmembrane domain, and a cytoplasmic serine/threonine kinase domain that mediates downstream signalling. The three-dimensional structure of the LRR-RK forms a twisted super helical shape (Fig. 5a), providing a large concave surface that accommodates specific peptide ligands such as bacterial flagellin (flg22) [35], [36].

A well-studied example of an LRR-RK is the *Flagellin Sensitive 2* (FLS2) receptor, which recognises flg22. When flg22 binds to FLS2, it induces the recruitment of a co-receptor, *Brassinosteroid Insensitive 1-associated receptor kinase* (BAK1). Structural analysis of the FLS2–flg22–BAK1 complex reveals that the flg22 peptide fits into a groove on the inner surface of the FLS2 LRR domain, with the C-terminal region of the LRR domain engaging in particularly strong interactions with the peptide (Fig. 5b). This binding facilitates the formation of a heterodimeric receptor complex, bringing FLS2 and BAK1 into proximity [37]. This tripartite complex enables trans-phosphorylation between FLS2 and BAK1 which activates downstream signalling cascades, including mitogen-activated protein (MAP) kinase pathways, upregulation of immune-related genes, and the production of reactive oxygen species. These responses collectively contribute to the plant’s innate immune defence against bacterial invasion [37].

#### 2.1.2 Molecular docking studies of protein-ligand and protein-protein interactions in plants

##### 2.1.2.1 Protein-ligand interactions

The structure of a protein can be used to perform molecular docking and analyse protein-ligand interactions [38], [39]. *In-silico* screening of extensive compound libraries can be performed to assess the affinity of protein-ligand binding. For example, Arabia *et al.* used molecular docking to analyse the interactions between curated sets of 9 rice Universal Stress Proteins (USP) and their predicted inhibitors showing that OsUSP32 and OsUSP33 exhibited high binding affinity to the inhibitor; “UspA inhibitor” (Zinc000104153710) and Protein Kinase C inhibitor luteolin [40]. Docking revealed the specific interactions, including hydrogen bonding and pi-pi interactions, providing insights into the binding mechanisms [40].

Supplementary Table 1 lists recent molecular docking studies that explored protein-ligand interactions involved in plant stress responses.

##### 2.1.2.2 Protein-protein interactions

**Fig. 6:**
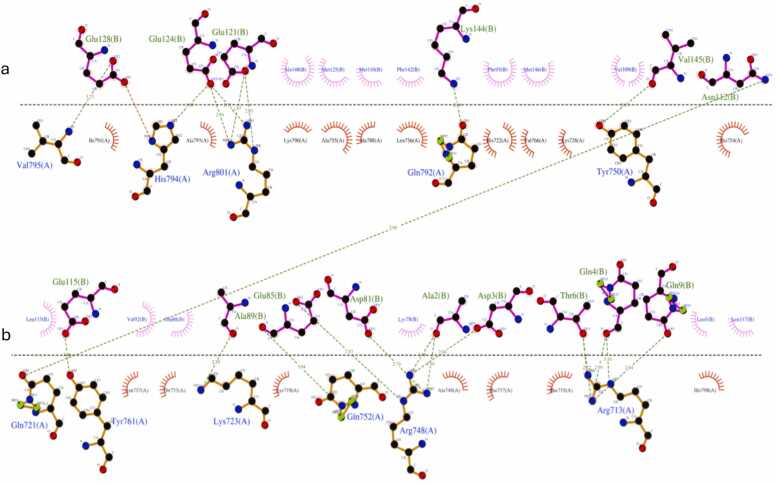
A LigPlot showing *Eleusine coracana* (Ec) CAMTA1 and Calmodulin interactions. Ec CAMTA1 are shown in (a) and Calmodulin (b). Hydrogen bonds are shown by green lines. Spiked arcs illustrate hydrophobic residues (This figure has been adapted from [41]). (For interpretation of the references to color in this figure legend, the reader is referred to the web version of this article.)

Structural modelling and docking analysis of CAlModulin-binding Transcription Activators (CAMTA) by Kadri *et al*., proteins involved in mediating abiotic stress responses in finger millet, provided valuable information about CAMTA-Ca^2 +^ -Calmodulin interactions with calcium [41]. These transcription activators are vital for several plant stress signalling pathways like response to cold, pathogens, and reactive Oxygen Species (ROS). Protein-protein docking revealed details of the interactions between CAMTAs and calmodulin (Fig. 6). This was further validated by analyses of the conserved residues.

### 2.2 Understanding the impacts of variants, paralogs and isoforms on protein structure and functions

Structural data is also important for investigating how protein function is impacted by mutations and varies in paralogs and isoforms [42]. The next sections report structural studies that reveal the impact of these modifications on protein structure and activity**.**

#### 2.2.1 Impact of variants

Structural analyses of genetic diversity can aid plant breeders to understand modifications that protect their crops and enhance yield. Resources such as the GWAS (Genome-Wide Association Studies) Atlas provide valuable information on SNPs for major crops such as rice, maize and soybean [43]. Understanding the location of non-synonymous SNPs (nsSNPs) and their associated mutations in coding regions, as well as their location in the 3D structure, is crucial for understanding their impact. Analyses of nsSNPs in protein domains have linked the associated mutations to both abiotic and biotic stress management in cotton [44]. Similar analyses in other plants also led to valuable insights. Computational pipelines have been used to map nsSNP data to protein structures. However, whilst resources exist for human nsSNPs, (e.g. SNP@Domain [45]), more efforts are needed for mapping nsSNPs to plant protein domains.

A recent study concerning nsSNPs in L-type lectin receptor kinases (LECRKs) used a combination of sequence and structure-based computational tools to analyse their impact on protein structure and function in *Arabidopsis thaliana* [46]. LECRKs are integral components of the plant stress signalling network, functioning as signal transduction sensors and mediators involved in both biotic and abiotic stress responses, including drought, salinity, cold, and innate immunity [47].

The analysis identified two nsSNPs, W431C and S415C, as having significant structural and functional consequences. Detailed structural analysis revealed that W431C disrupts a network of critical hydrogen bonds within the kinase domain, leading to the formation of a cavity in the protein core, ultimately destabilising the protein structure. The mutant is unable to interact with SRK2C (SNF-1 related protein kinase 2 C), a drought signalling protein. This loss of interaction is supported by a marked increase in the distance between the centre of mass of the docked mutant and SRK2C proteins, compared to the wild-type complex [46]. Similarly, the S415C mutation alters the chemical properties of a residue located within the kinase domain, disturbing local hydrogen bonding interactions and potentially impairing proper protein folding. Collectively, these results suggest that the mutations undermine LECRK's role in mediating plant responses to environmental stresses.

Another study by Bhardwaj *et al*. analysed the functional consequences of nucleotide polymorphisms in wild *(Solanum habrochaites)* and cultivated (*Solanum lycopersicum*) species of tomato [48]. A total of 1838 nsSNPs were identified from publicly available Expressed Sequence Tag (EST) and Next Generation Sequence (NGS) data of both species. The identified nsSNPs represent naturally occurring variations between these two species. The authors performed a structural analysis of the HMG1 (High Mobility Group box 1) protein, which is involved in DNA regulatory processes and is part of the base excision repair pathways, including the repair of damage due to oxidative stress. It is also known for its interaction with the p53 ligand. A deleterious nsSNP identified in HMG1 results in a Tyr416Asp amino acid change which affects the binding of the ligand.

**Fig. 7:**
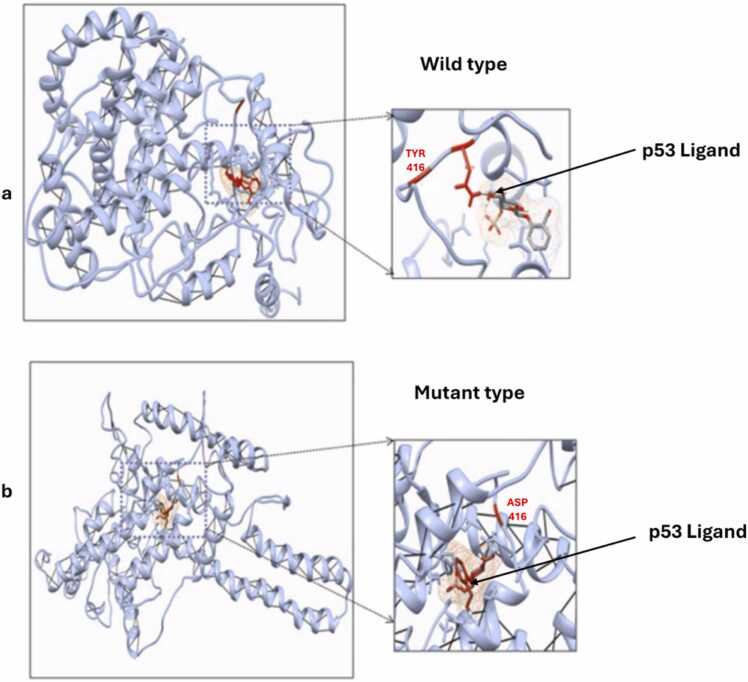
Pictorial representation of native and mutant models of HMG1 protein. (a) the p53 ligand binds native HMG1 close to SNP site with an energy of −273.8 kcal/mol (b) p53 ligand binds mutant HMG1 at a site remote from the native site with an energy of −267.4 kcal/mol. Adapted from [48].

Docking simulations with the p53 revealed that in the wild HMG1–p53 complex, the ligand binds appropriately at the active site near residue 416 Tyr (Figs. 7a and 7b). In contrast, the Tyr416Asp mutation in the new strain induced a conformational change in the site that shifted the P53 ligand binding to a distant site, away from the original pocket (Figs. 7a and 7b) potentially impairing the function of the protein [48].

Additionally, a recent study [49] by Hernández *et al.* studied the impact of mutations on the binding of the G-protein coupled receptor-1 (GPCR1) in *Arabidopsis thaliana* following the phytohormones such as abscisic acid (ABA) and gibberellin A1 (GA1). Structures of GPCR1 were obtained by homology modelling and mutations introduced, followed by energy minimisation. Subsequently, docking studies of ABA and GA1 to the receptor were performed. Molecular dynamics (MD) simulations were also conducted across varying temperature and pressure conditions to evaluate stability and binding changes. This approach identified mutations in charged residues that decreased the stability of apo-GPCR1, as well as the complexes with ABA and GA1, by disrupting salt linkages, thus highlighting residue interactions necessary for receptor stabilization and function [49].

GPCR1s have 6 transmembrane helices (TMs) named TM1–6. Mutations within interhelical regions such as TM2 −TM4, TM3 −TM4, and TM5 −TM6, including those affecting interhelical hydrogen bonds, were predicted to disrupt the structure of GPCR1, affecting internal interactions and stability. Analyses performed by Hernández et al. [49] identified key stabilizing interactions in the native form, which when mutated to nonpolar residues like glycine, alanine or phenylalanine, induced significant destabilization of the helix packing, shedding light on major stabilising interactions. Such predictions serve as a valuable starting point for experimental validation and further understanding of GPCR1's function in plants.

#### 2.2.2 Impact of paralogs

Paralogous proteins emerge following a gene duplication event within the genome and subsequently evolve, undergoing mutations that may lead to distinct functional behaviour or involvement in different pathways [50]. An important stress-modulating pathway in plants is the Mitogen-Activated Protein Kinase (MAPK) pathway, containing both kinases and phosphatases that regulate diverse cellular functions. A structure-based study by Yu *et al.* focused on *Chrysanthemum morifolium* and found that changes in residues of three paralogous phosphatase proteins, CmDsPTP1-LIKE1/2/3, affected how these proteins dephosphorylated four groups of MAPKs in chrysanthemums. Variations in these paralogues (M87V, T277P and V6L) changed their interactions with kinases as they altered the surface interface properties, allowing the proteins to bind different kinase partners more effectively [51].

Various studies exploiting pathway analyses showed how members of a multidrug and toxin gene family (MATE) facilitated increased uptake of aluminium during stress [52]. These proteins capture aluminium in the form of aluminium citrate and transport it to the vacuoles to be discarded, hence reducing toxicity. The Citrate-Exuding Motif (CEM) in these proteins has a vital role in cation transport. Moreover, Aluminium citrate produced in the root cells is transported to the leaves by the help of TaMATEs to be deposited in the vacuoles, thus lessening Al toxicity [53].

Analyses of 6 members of the family used structural data to characterize their interaction with the citrate moiety of aluminium citrate. Sitemap analysis of the docked proteins with their ligands revealed several binding sites in the central cavity between the N and C-terminal domains of wheat TaMATE proteins [54]. Furthermore, it has been shown that the conserved CEM motif from TaMATE proteins is associated with diverse citrate binding sites in different paralogs (TaMATE4, 9, 15, 74, 85 and 93 have 11, 13, 12, 14, 11, and 15 binding sites, respectively).

Structural insights into functional variations in paralogous proteins are illustrated by phytochromes, which play a crucial role in helping plants perceive and respond to environmental stress (e.g., salt, drought, and temperature) by modulating gene expression and hormonal signalling pathways. They act as light-sensitive regulators that integrate external light cues with internal stress responses to enhance plant adaptation and survival [42], [55]. For instance, Phytochromes A (PhyA) and B (PhyB) in *Arabidopsis thaliana* are closely related red/far-red light photoreceptors (50 % sequence identity) with similar domain structures but different functions [56]. While both possess modular assemblies consisting of photosensory modules (PSMs) for detecting light conserved Per–Arnt–Sim (PAS) domains for dimerization assembly, and histidine kinase-related domains (HKRDs) involved in signal transmission, their dimeric forms vary [57].

Both PhyA and PhyB possess the same classic tripartite structure in their PSM module: an N-terminal PAS (nPAS) domain that facilitates dimeric assembly, a GAF (cGMP phosphodiesterase/adenylate cyclase/FhlA) domain responsible for chromophore binding, and a phytochrome (PHY) domain for light reception and photoconversion. Together, these coordinate the phytochromobilin (PΦB) chromophore, enabling the perception of red and far-red light [56]. However, despite this conserved domain arrangement, their internal geometries differ. In PhyA, the PHY domains are symmetrically positioned over the nPAS–GAF bi-domain, whereas in PhyB they are displaced, causing a ∼17° deviation in the trajectory of the helical spine, as depicted by the structural study illustrated in Figs. 9a and 9b. This displacement results in changes in the structural arrangement of the dimer and leads to a more asymmetric and less stable photosensory platform in PhyB [56].

These structural differences have functional consequences. PhyA is optimised for activity under low-light and thermally unstable conditions, while PhyB is designed for rapid responses to dynamic light environments. Thermal reversion studies reveal that PhyA remains in its active state longer than PhyB, indicating greater thermal stability. Furthermore, the PAS–nPAS–GAF interdomain interface plays an important role in maintaining dimer stability in PhyB than in PhyA [57]. Mutational analyses further support this: for example, the I823E substitution significantly disrupts PhyB dimerization and associated function, whereas it has only a minor impact on PhyA [56].

Together, these structural and functional differences reflect evolutionary adaptations that enable PhyA and PhyB to specialize in different aspects of light sensing. These adaptations highlight the sophisticated role of phytochromes in helping plants sense and respond to their environment [56].

#### 2.2.3 Impact of Isoforms

**Fig. 8:**
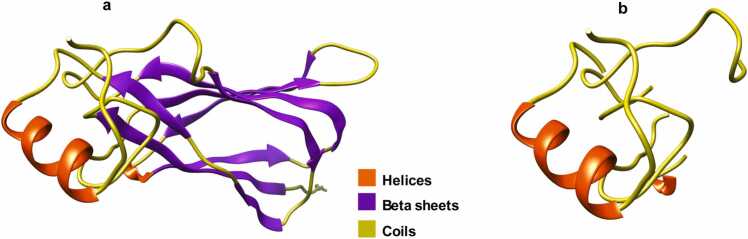
Alternatively spliced isoform of Ole e1 (P19963) protein in *Gossypium arboreum* (a) complete structure containing the beta sheets (b) structure without beta sheets (the image was based on the observations reported in [60], recreated using AlphaFold [12] and visualized using UCSF Chimera [20]).

Exons can combine in different ways to produce diverse protein isoforms, each with unique functions[58]. Isoforms can also be generated by intron retention [59]. For example, a structural study of the alternatively spliced isoform of Ole e1 (P19963) protein in *Gossypium arboreum* (tree cotton) revealed that it had a disrupted protein structure and had lost the beta sheets forming the beta sandwich required for its functional role as a toxin (see Figs. 8a and 8b) [60].

**Fig. 9:**
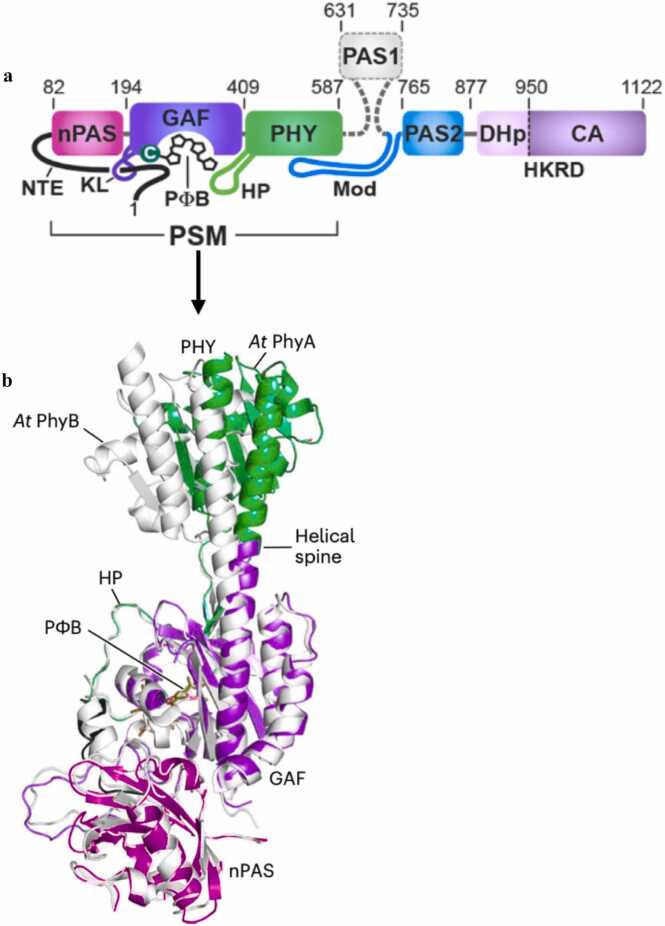
a) scheme showing Phy A and PhyB domain organisation b) Structure illustrating differences in the orientations of the domains in PSM module; a Per/Arnt/Sim (nPAS) domain-cGMP-specific phosphodiesterase/adenylyl cyclase/FhlA (GAF) domain and a Phy-specific (PHY) from PhyA (coloured), superimposed onto PhyB (PDB ID 8F5Z and 7RZW). The nPAS, GAF, helical spine, phytochromobilin (PΦB), and hairpin (HP) regions are shown. The figure has been adapted using data from [56]. (For interpretation of the references to color in this figure legend, the reader is referred to the web version of this article.)

## 3 Advances in structure prediction

AI-powered protein structure prediction has revolutionized the field of biology, enabling researchers to determine the structures of millions of proteins rapidly and accurately. In 2019, CASP assessment (CASP14), a biannual event for protein structure prediction evaluation, DeepMind’s neural network-based AlphaFold2 methods outperformed other approaches, achieving high accuracy for most of the targets (approximately 90 %) in modelling protein backbone and side chains, with a TM-score of 244.0, significantly higher than the next best group with TM-score of (98.0) [61].

AlphaFold2 is an attention-based deep learning model trained end-to-end to predict the structure of a protein. Protein folding entails interactions between amino acids that may be sequentially distant yet proximal in 3D arrangement, and the attention mechanism and end-to-end training allow the model to adeptly capture such interactions. AlphaFold2 was trained using a large repository (the Protein Data Bank) of protein structural data [62]. Its neural network exploits various inputs including a multiple sequence alignment providing evolutionary information and structural homologues from the PDB. Additionally, the model uses a 2D matrix that represents the likelihood of interactions between pairs of amino acids within the protein. The output is fed back into the network three times, iteratively refining the structure prediction [12]. For a detailed review on the topic, please see [63], [64].

The AlphaFold Protein Structure Database currently provides protein structure models for a total of 214 million protein sequences in UniProt [65]. The models can be evaluated based on their plDDT score (per-residue scores on the lDDT-Cα metric) and PAE (predicted aligned error) value. The plDDT score gauges per-residue confidence, while the PAE value assesses overall protein topology and domain packing confidence [12]. Recently, AlphaFold3 has been introduced as a newer version of AlphaFold2 but the main utility of this tool is in the prediction of protein complexes and this remains to be more widely explored [15].

Another protein structure prediction method, RoseTTAFold2 employs a unique three-track neural network architecture that analyses the protein sequences, the interactions between amino acids, and the potential 3D structures, simultaneously [13]. AlphaFold2 outperformed RoseTTAFold2 in test cases [66]. However, the more recent RoseTTAFold All-Atom offers protein-structure prediction accuracy on par with AlphaFold2, achieving a median global distance test (GDT) score of 85 compared to AlphaFold2's 86 [67]. RoseTTAFold All-Atom has the ability to model interactions between proteins and small molecules, as well as covalent modifications to proteins. It can also model complexes involving proteins and multiple non-protein molecules. This feature is useful for designing proteins that bind to small molecules and for designing sensors [67].

Another protein structure prediction tool, OmegaFold [68], which uses protein language models, is capable of predicting the structure of orphan proteins having no homologues, from a single sequence. OmegaFold is approximately 10 times faster than AlphaFold, albeit with slightly lower accuracy. A related tool ESMfold [14] has further accelerated the provision of high-resolution structure predictions from sequences. The developers trained their AI-based models using 15 billion parameters (the largest language model for proteins to date) and the UniRef sequence database [69]. This method is sixty-fold faster than AlphaFold2, albeit with slightly lower accuracy. The ESM Metagenomic Atlas, based on this approach, contains ∼617 million 3D models for proteins in the MGnify resource [70], shedding light on a vast array of previously structurally uncharacterized protein sequences from metagenomes [14].

In CASP 15, most research groups employed AlphaFold2, but often in combination with their own tools. Some groups improved alignment quality through using more expansive databases or methods like DeepMSA2 that improved the alignment quality [71]. These alignments were then fed into AlphaFold2 (or trRosetta in some cases) to generate model predictions [72], [73]. Researchers also employed strategies such as dropouts or disabling pairing (where randomly selected nodes in a neural network are temporarily deactivated during training iterations to prevent overfitting) and created numerous models for every target. Template searching enhancements were also explored, exploiting HHsearch or HMMsearch, followed by novel scoring schemes to select the best models [74], [75]. It was concluded that the average TM score of AlphaFold2 can be increased from 0.7 to 0.8, resulting in models even more closely aligned with experimental structures [76].

AlphaFold-multimer, also developed by the DeepMind team, uses sequence information to predict accurate multimeric structures [77]. Furthermore, AlphaMissense, a recently developed tool, builds on the AlphaFold architecture and is trained on variant data. It combines sequence embeddings from unsupervised protein language models together with population frequency data to predict the pathogenicity of missense mutations (single amino acid changes in proteins) [78].

**Fig. 10:**
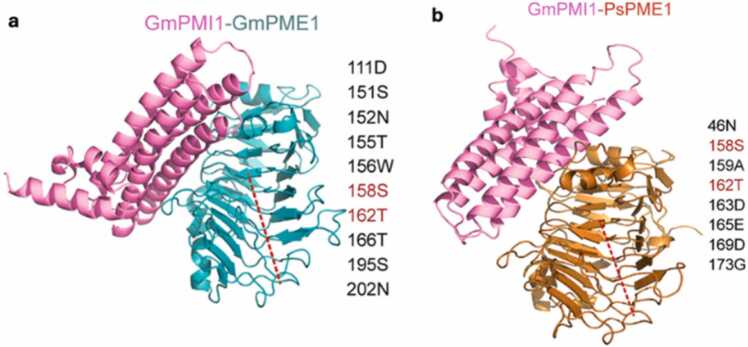
Colour-coded complexes of (a) Soybean GmPME1 and (b) fungal PsPME1 with soybean GmPMI1, as predicted by AlphaFold2-Multimer. (Figure has been adapted from [79]). (For interpretation of the references to color in this figure legend, the reader is referred to the web version of this article.)

The application of AlphaFold2 to uncharacterised plant proteins is already starting to yield interesting insights. For instance, a structural study [79] of pathogen Pectin methyltransferases (PMEs) which degrade pectin in the plant cell wall, and their inhibitors (PMIs), produced by plants particularly in soybean (*Glycine max)* has enhanced our understanding of protein interactions at the molecular level for this important protein. PMI is responsible for resisting pectin degradation under fungal attack. AlphaFold2-Multimer was used to model the interactions between GmPMI1 from soybean and both PsPME1 (pathogen PME) and GmPME1 (soybean PME), revealing distinct binding interfaces for the interactions involving the host interactor or the pathogen interactor (Figs. 10a and 10b). This enabled the identification of specific residues for targeted mutation to engineer GmPMI1R, which selectively inhibits PsPME1 while sparing GmPME1. Modelling revealed that mutations to more polar residues in the interface aided in PMI PME (pathogen) binding and could potentially enhance resistance against fungal pathogens [79]. In contrast to its natural counterpart GmPMI1, GmPMI1R (mutated PMI from soybean) contained mutations of nine amino acid residues (D102A, G104A, D111A, S151k, N152F, T155D, W156G, S195A, N202A).

Some other plant-based studies exploiting recent structure prediction tools are listed in Table 1.

**Table 1:** Recent studies employing AI-based structure prediction tools.

No.	Plant Species	Tools used	Protein and related stress factor	Key Takeaway	Reference
1	Tomato	AlphaFold2	*Tsw*, a protein associated with viral resistance	An extended LRR domain with helical turns enables specific pathogen recognition and binding, enhancing immune specificity.	[80]
2	Arabidopsis	AlphaFold2	Aspartate Protease APCB1 involved in defense against fungal pathogens	A surface loop gates substrate access, aiding pathogen response.	[81]
3	Arabidopsis	AlphaFold2	Secreted Aspartate Proteases (SAP1) with antibacterial activity	Active site motifs (DLGG, SSVN) with unique bonding patterns that may enable cleavage of the bacterial protein MucD.	[82]
4	Arabidopsis	AlphaFold2	Peroxidase (PER) involved in biotic and abiotic stress regulation	Two isoforms; closed mouth PER, stress-induced, restricts large substrates via Alpha/Beta motifs; open mouth PER, active in normal conditions, lacks H-bonds, allowing broad substrate oxidation.	[83]
5	*Sorghum bicolor*	RoseTTaFold & AlphaFold2	RNA polymerase II-associated protein 3 (sbRPAP 2) involved in abiotic stress management	Identification of residues involved in interaction with heat shock proteins (Hsp90, Hsp70).	[84]
6	Arabidopsis	OmegaFold & AlphaFold2	Type I and II MADS transcription factors (TFs) involved in abiotic stress management	Type II TFs structurally resemble human homologs and bind DNA flexibly in stress response. Type I TFs lack a kink in the second helix, suggesting a new phylogeny, representing a divergent mechanism in land plant stress adaptation.	[85]
7	Arabidopsis	AlphaFold3	β-amylases (BAM9) and ɑ-amylase (AMY3) responsible for stress induced starch catabolism	BAM9 is a compact globular protein, while AMY3 is a flexible dimer with an α-hairpin domain enabling complex formation. The BAM9–AMY3 complex likely activates AMY3 for starch degradation, supplying energy during stress recovery.	[86]

AI-based tools like AlphaFold2 demonstrate significant potential for structurally characterising protein functions. However, challenges remain that should be addressed to increase their reliability. One issue stems from bias in the training data extracted from the PDB, which only contains proteins amenable to crystallisation or NMR studies and lacks, for example, transmembrane proteins [87]. Additionally, updating prediction databases like the AlphaFold Database is computationally intensive raising concerns about scalability and feasibility as the protein sequence datasets like UniProt and metagenome resources like MGnify continue to expand exponentially [88]. Furthermore, the ability to accurately predict folding pathways or structural dynamics remains limited [87]. Future improvements will require careful curation of test datasets and standardized evaluation protocols to mitigate computational and environmental burdens [87].

Despite these challenges, it is clear that significant expansions in the structural coverage of plant proteins by AlphaFold and the continuing developments in protein structure prediction have ushered in a new era in structural analyses of plant proteins associated with stress resistance. The ability to accurately capture the three-dimensional arrangements of binding pockets and protein interfaces will enable the rational design and associated protein engineering of plant proteins associated with stress response in order to increase resilience to stress.

Although, there are few such studies in the literature to date, due to the paucity of experimental protein structures, it is likely that the new predicted structure data will facilitate these efforts going forward. As many of the examples presented in this review have demonstrated, protein structures are valuable for identifying the key residues in binding pockets, active sites, protein interfaces and allosteric sites (e.g. residues in the secondary shell of the active and binding sites) which may also contribute to protein activity. These examples illustrate the power of structure-based analyses to suggest residue mutations that could be engineered to enhance the protein function.

## 4 Conclusion

By employing diverse structural analyses, including mapping of GWAS SNPs to structures, exploring variants in paralogs and isoforms, and molecular docking followed by molecular dynamic simulations, scientists can identify and explain specific genetic variants and changes in paralogues and isoforms that confer improved stress tolerance and resilience in plants. Structure-based analyses also enable protein engineering for enhancing specific stress-related functions. Recent advances in protein structure prediction, such as AlphaFold2, OmegaFold, ESMFold, RoseTTAFold All-Atom, AlphaFold-Multimer, and AlphaFold3 will allow researchers to gain comprehensive insights into the 3D structures of stress-related proteins and expand our understanding of the functional mechanisms involved in plant stress management. This new wealth of protein structures holds great promise for developing crop strains with higher yields, increased resistance to environmental stressors, and improved agricultural sustainability, marking the advent of a new era of precision agriculture.

## Author contributions

**Fatima Shahid** and **Su Datt Lam** conceptualized the study. **Su Datt Lam, Christine Orengo, Nurulhikma Md Isa**, and **Nyuk Ling Ma** performed the funding acquisition. Fatima Shahid wrote the original draft, conducted the literature search, and prepared the figures. **Neeladri Sen** validated the study and reviewed and edited the manuscript. **Hawa Najibah Rasni** assisted in the literature search and revision of the manuscript. **Nurulhikma Md Isa** and **Nyuk Ling Ma** provided valuable feedback to correlate structural knowledge to plant biology. **Christine Orengo** and **Su Datt Lam** supervised the study and edited the manuscript. All authors contributed to the article and approved the submitted version.

## CRediT authorship contribution statement

**Hawa Najibah Rasni:** Writing – review & editing. **Nyuk Ling Ma:** Validation. **Nurulhikma Md Isa:** Validation. **Su Datt Lam:** Writing – review & editing, Supervision, Project administration, Funding acquisition, Conceptualization. **Christine Orengo:** Writing – review & editing, Validation, Supervision, Funding acquisition. **Neeladri Sen:** Writing – review & editing. **Fatima Shahid:** Writing – review & editing, Writing – original draft, Visualization, Validation, Resources, Methodology, Investigation, Funding acquisition, Formal analysis, Data curation, Conceptualization.

## Ethical considerations

Not Applicable.

## Funding statement

We want to thank the Fundamental Research Grant Scheme from the Ministry of Higher Education Malaysia (grant number: FRGS/1/2024/STG01/UKM/02/2) for funding this work. NS would like to thank 10.13039/501100000268BBSRC funding BB/S020144/1.

## Declaration of Generative AI and AI-assisted technologies in the writing process

During the preparation of this work, the authors used NotebookLM in order to cross-check the stated facts from the literature. After using this tool/service, the authors reviewed and edited the content as needed and take full responsibility for the content of the publication.

## Declaration of Competing interest

The author(s) declared no potential conflicts of interest with respect to the research, authorship, and/or publication of this article.

## Data Availability

Not applicable.

## Footnotes

Appendix A Supplementary data associated with this article can be found in the online version at doi:10.1016/j.csbj.2025.07.021.
