# Supplementary material for: Review of protein structure-based analyses that illuminate plant stress mechanisms
Source: Comput Struct Biotechnol J. 2025 Jul 13;27:3155–66. doi: 10.1016/j.csbj.2025.07.021 (PMC12302779; doi:10.1016/j.csbj.2025.07.021)
Supplement: Supplementary file 1 — Supplementary material [file mmc1.docx]

**Supplementary Table 1:** Recent molecular docking studies exploring protein–ligand interactions in plant proteins associated with stress response.

| **Plant Species** | **Protein** | **Ligand** | **Key Outcomes** | **Reference** |
| --- | --- | --- | --- | --- |
| Maize | Glycosyltransferase (UGT706F8) | ACCA (1-aminocyclopropane-1-carboxylic acid) | ACCA binds to UGT706F8 via π- and van der Waals interactions, suggesting stress response mechanism. | (1) |
| Empress tree | Squamosa Promoter-Binding Protein-Like (PfSPL) Protein | Methyl Methanesulfonate (MMS) and Rifampicin (Rif)) | MMS and Rif bind to SBP domains via hydrogen bonds; may activate PfSPLs, suppressing phytoplasma infection and reversing disease symptoms. | (2) |
| Sugarcane (*E. arundinaceus, S. officinarum,* hybrid) | α-Expansin 1 (EXPA1) | Xylose | Hydrogen bonding with xylose may facilitate cell wall relaxation and flexibility under abiotic stress; key residues identified for each variety. | (3) |
| Wheat | Fatty Acid Desaturases (TaFAD2.6 and TaFAD2.8) | Oleic acid | Oleic acid interacts via hydrogen bonds and hydrophobic contacts near histidine-rich active site regions supporting  essential role of conserved motifs in ligand binding and function. | (4) |
| Barrel medic *(M. truncatula)* | Glutathione S-transferase (MtGSTU17) | Glutathione (GSH) and CDNB | MtGSTU17 binds GSH through six hydrogen bonds and one hydrophobic interaction; binds CDNB through four hydrogen bonds and one π–σ interaction; reveals conservation of substrate binding in GSTs. | (5) |
| Wheat | MYB Transcription Factor | 2,6-ditert-butylcyclohexa-2,5-diene-1,4-dione; 3,5-ditert-butylphenol | Ligands show strong binding to MYB protein via hydrogen, π-alkyl, and π-sulfur bonds suggesting that inhibition of MYB may enhance drought tolerance. | (6) |

1. Debnath S, Elgorban AM, Bahkali AH, Eswaramoorthy R, Verma M, Tiwari P, et al. Exploring the efficacy of 1-amino-cyclopropane-1-carboxylic acid (ACCA) as a natural compound in strengthening maize resistance against biotic and abiotic stressors: an empirical computational study. Frontiers in Microbiology. 2023;14:1232086.

2. Yang H, Zhai X, Zhao Z, Fan G. Comprehensive analyses of the SPL transcription factor family in Paulownia fortunei and their responses to biotic and abiotic stresses. International Journal of Biological Macromolecules. 2023;226:1261-72.

3. Narayan JA, Dharshini S, Manoj V, Padmanabhan TS, Kadirvelu K, Suresha G, et al. Isolation and characterization of water-deficit stress-responsive α-expansin 1 (EXPA1) gene from Saccharum complex. 3 Biotech. 2019;9:1-13.

4. Hajiahmadi Z, Abedi A, Wei H, Sun W, Ruan H, Zhuge Q, et al. Identification, evolution, expression, and docking studies of fatty acid desaturase genes in wheat (Triticum aestivum L.). BMC genomics. 2020;21:1-20.

5. Hasan MS, Singh V, Islam S, Islam MS, Ahsan R, Kaundal A, et al. Genome-wide identification and expression profiling of glutathione S-transferase family under multiple abiotic and biotic stresses in Medicago truncatula L. PLoS one. 2021;16(2):e0247170.

6. Paul GK, Mahmud S, Dutta AK, Sarkar S, Laboni AA, Hossain MS, et al. Volatile compounds of Bacillus pseudomycoides induce growth and drought tolerance in wheat (Triticum aestivum L.). Scientific Reports. 2022;12(1):19137.
